# New Results on Passivity Analysis of Stochastic Neural Networks with Time-Varying Delay and Leakage Delay

**DOI:** 10.1155/2015/389250

**Published:** 2015-08-05

**Authors:** YaJun Li, Zhaowen Huang

**Affiliations:** Department of Electronics and Information Engineering, Shunde Polytechnic, Foshan 528300, China

## Abstract

The passivity problem for a class of stochastic neural networks systems (SNNs) with varying delay and leakage delay has been further studied in this paper. By constructing a more effective Lyapunov functional, employing the free-weighting matrix approach, and combining with integral inequality technic and stochastic analysis theory, the delay-dependent conditions have been proposed such that SNNs are asymptotically stable with guaranteed performance. The time-varying delay is divided into several subintervals and two adjustable parameters are introduced; more information about time delay is utilised and less conservative results have been obtained. Examples are provided to illustrate the less conservatism of the proposed method and simulations are given to show the impact of leakage delay on stability of SNNs.

## 1. Introduction

During the past several decades, neural networks have gained great attention because of their potential application in pattern classification, reconstruction of moving image, and combinatorial optimization. In addition, time delay is a natural phenomenon frequently encountered in various dynamic systems such as electronic, chemical systems, long transmission lines in pneumatic systems, biological systems, and economic and rolling mill systems. Delays in neural networks can cause oscillation, instability, and divergence, which are very often the main sources of poor performance of designed neural networks. So the stability analysis and state estimation of neural networks with various time delays have been widely investigated by many researchers; see [1–8] and the references therein.

Furthermore, when modeling real nervous systems, stochastic disturbance is one of main resources of the performance degradations when applying the neural networks, because the synaptic transmission is a noisy process introduced by random fluctuation from the release of neurotransmitter and other probabilistic causes. In recent years, the stability analysis for stochastic neural networks with time delay has become a hot research topic; by virtue of various inequality technics and *M*-matrix theory, many important research results about neural networks with different type of time delays, such as constant delay, time-varying delay, or distributed delay, have been reported; see, for example, [8–14] and the references therein.

The passivity theory, which originated from circuit theory, plays an important role in the analysis of stability of linear or nonlinear systems. The main character of passivity theory is that the passive properties of a system can keep the system internally stable. Because it is a very effective tool in studying the stability of uncertain or nonlinear systems, the passivity theory has been used widely in fuzzy control [15], complexity [16], synchronization [17], signal processing [18], and adaptive control [19].

Recently, based on the Lyapunov-Krasovskii theory, passivity and dissipativity analysis of neural networks with various delays and uncertainties have been discussed and many interesting results have been reported [20–28].

In [29–35], based on the Lyapunov-Krasovskii, LMI method, and a delay fractioning technique, the passivity and robust passivity of stochastic neural networks with delays and uncertainties have been studied; some sufficient conditions on the passivity of neural networks with delays have been obtained. In [31], authors investigated passivity of the stochastic neural networks with time-varying delays and parameters uncertainties by applying free-weighting matrix and the lower conservatism results are obtained by comparing with the existing results.

On the other hand, in many practical problems, a typical time delay called leakage delay or forgetting delay exists in dynamical system, which has a tendency to destabilize the system; it has been one of the research hot topics recently and many research achievements have been reported [20, 36–42].

As pointed out in [36], neural networks with leakage delay are a class of important neural networks, and time delay in the leakage term also has great impact on the dynamics of neural networks; sometimes it has more significant effect than other kinds of delays on dynamics of neural networks; the stability analysis of neural networks system involving leakage delay has been researched extensively; see, for example, [37–40] and the references therein. Very recently, in [42], by virtue of free weight matrix and LMIs method, the passivity problem for a class of stochastic neural networks with leakage delay is studied; the sufficient condition making the system passive is presented, but leakage delay under consideration is a constant; but, in practical dynamical systems, the leakage delay can be time-varying, which is often more general and complex than leakage delay being a constant. To the best of authors' knowledge, no research results have been reported about the condition that leakage delay is time-varying, which motivates our idea.

Motivated by the aforementioned discussions, this paper focuses on the passivity problem for a class of stochastic neural networks (SNNs) system with time-varying delay and leakage delay; by constructing a new Lyapunov functional, a set of sufficient conditions are derived to ensure the passivity performance for a class of stochastic neural networks with time-varying delays and leakage delay. By virtue of the delay decomposition idea [8], combining with some integral inequality technic [7], or free-weighting matrix approach [9, 26], two adjustable parameters are introduced and made full use of. All results are established in the form of LMIs and can be solved easily by using the interior algorithms, which can be efficiently solved by Matlab LMI Toolbox and no tuning of parameters is required. Finally, numerical examples are given to demonstrate the effectiveness and less conservatism of the proposed approach.

The main contributions of this paper are summarized as follows:

- The leakage delay studied is time-varying, so the research model is more general and complex than that in [42].
- The neuron activation function is assumed to satisfy sector-bounded condition, which is more general and less restrictive than Lipschitz condition, so the less conservatism results can be expected.
- The derivative of time-varying can be extended to be more than 1.
- How the leakage delay affects the stability result is discussed.

*Notation*. Throughout this paper, if not explicit, matrices are assumed to have compatible dimensions. The notation *M* > (≥, <, ≤)  0 means that the symmetric matrix *M* is positive-definite (positive-semidefinite, negative, and negative-semidefinite). *λ*_min_(·) and *λ*_max_(·) denote the minimum and the maximum eigenvalue of the corresponding matrix; the superscript “*T*” stands for the transpose of a matrix; the shorthand diag{⋯} denotes the block diagonal matrix; ‖·‖ represents the Euclidean norm for vector or the spectral norm of matrices. *I* refers to an identity matrix of appropriate dimensions. *𝔼*{·} stands for the mathematical expectation; *∗* means the symmetric terms. Sometimes, the arguments of a function will be omitted in the analysis when no confusion can arise.

## 2. System Description

Consider the SNNs with time-varying delay as follows:

(1) dxt=−Axt−δt+W0fxt+W1fxt−τt+utdt+σxt,xt−τt,utdωt,yt=fxt,

where *x*(*t*) = [*x*_1_(*t*), *x*_2_(*t*),…, *x*_*n*_(*t*)]^*T*^ ∈ *ℝ* ^*n*^ is the neural state vector and *u*(*t*) = [*u*_1_(*t*), *u*_2_(*t*),…, *u*_*n*_(*t*)]^*T*^ is the input. *y*(*t*) = [*y*_1_(*t*), *y*_2_(*t*),…, *y*_*n*_(*t*)]^*T*^ ∈ *ℝ* ^*n*^ is the output; *W*_0_, *W*_1_ ∈ *ℝ* ^*n*^ are the connection weight matrix and the delayed connection weight matrix, respectively; *A* = diag⁡(*a*_1_, *a*_2_,…, *a*_*n*_) is a positive diagonal matrix; *f*(*x*(*t*)) = [*f*_1_(*x*(*t*)), *f*_2_(*x*(*t*)),…, *f*_*n*_(*x*(*t*))]^*T*^ ∈ *ℝ* ^*n*^ is the neuron activation function with *f*(0) = 0; *n* denotes the number of neurons in neural networks; *ω*(*t*) = [*ω*_1_(*t*), *ω*_2_(*t*),…, *ω*_*m*_(*t*)]^*T*^ ∈ *ℝ* ^*m*^ is an *m*-dimension Brownian motion defined on a complete probability space (*Ω*, *ℱ*, *P*), satisfying

(2) Edωt=0,Edω2t=dt.

*τ*(*t*) is the transmission delay and is assumed to satisfy

(3) 0τt≤τ,−μτ˙t≤μ.

*δ*(*t*) is the leakage delay that satisfies

(4) δt≤δ,δ˙t≤ρδ,

where *τ*, *μ*, *δ*, *ρ*_*δ*_ are some positive scalar constants.

**Assumption 1 .**

For *i* ∈ {1,2,…, *n*} and ∀*x*, *y* ∈ *ℝ*, *x* ≠ *y*, the neuron activation function *f*(·) is continuous and bounded and satisfies

(5) fx−fy−Λ1x−yT·fx−fy−Λ2x−y<0,

where Λ_1_ and Λ_2_ are some constant known matrices.

**Remark 2 .**

In this paper, the above assumption is made on neuron activation function, which is called sector-bounded neuron activation function. When Λ_1_ = Λ_2_ = −Λ, condition (5) becomes

(6) fx−fyTfx−fy≤x−yTΛTΛx−y.

So it is less restrictive than the descriptions on both the sigmoid activation functions and the Lipschitz-type activation functions.

**Assumption 3 .**

There exist three constant matrices Σ_1_, Σ_2_, and Σ_3_ such that

(7) σxt,xt−τt,ut2≤Σ1xt2+Σ2xt−τt2+Σ3ut2.

**Definition 4 (see [22]).**

The delayed SNNs are said to stochastically passive if there exists a scalar *γ* ≥ 0 such that

(8) $$\begin{array}{c} 2E\overset{t}{\underset{0}{\int }}y^{T}\left(\;s\;\right)u\left(\;s\;\right)ds\geq -\gamma E\overset{t}{\underset{0}{\int }}u^{T}\left(\;s\;\right)u\left(\;s\;\right)ds \end{array}$$

for all *t* ≥ 0 and for all solution of (1) with *x*(0) = 0.

**Remark 5 .**

The different output equation can lead to different definitions. In [31, 42], the output equation expression is *y*(*t*) = *f*(*x*(*t*)) and *y*(*t*) = *Gf*(*x*(*t*)), respectively. In order to compare our result with that in [42], we take *G* = *I*, so we have the same definition as that in [42].

At first, we give the following lemmas which will be used frequently in the proof of the our main results.

**Lemma 6 (see [4]).**

For any constant symmetric positive defined matrix *J* ∈ *ℝ* ^*m*×*m*^, scalar *η*, and the vector function *ν* : [0, *η*] → *ℝ* ^*m*^, the following inequality holds:

(9) $$\begin{array}{c} \eta \overset{\eta }{\underset{0}{\int }}\nu^{T}\left(\;s\;\right)J\nu \left(\;s\;\right)ds\geq {\left(\;\overset{\eta }{\underset{0}{\int }}\nu \left(\;s\;\right)ds\;\right)}^{T}J\left(\;\overset{\eta }{\underset{0}{\int }}\nu \left(\;s\;\right)ds\;\right). \end{array}$$

**Lemma 7 (see [5]).**

For given proper dimensions constant matrices Φ_1_, Φ_2_, and Φ_3_, where Φ_1_^*T*^ = Φ_1_ and Φ_2_^*T*^ = Φ_2_ > 0, we have Φ_1_ + Φ_3_^*T*^Φ_2_^−1^Φ_3_ < 0 such that only and only if

(10) Φ1Φ3T∗−Φ2<0,or  −Φ2Φ3∗Φ1<0.

**Lemma 8 (see [7]).**

For given function *τ*(*t*) satisfying $\mu_{1}\leq \dot{\tau }(t)\leq \mu_{2}$, there exist nonnegative functions *λ*_1_(*t*) ≥ 0 and *λ*_2_(*t*) ≥ 0 satisfying *λ*_1_(*t*) + *λ*_2_(*t*) = 1 such that the following equation holds:

(11) $$\begin{array}{c} \dot{\tau }\left(\;t\;\right)=\mu_{1}\lambda_{1}\left(\;t\;\right)+\mu_{2}\lambda_{2}\left(\;t\;\right). \end{array}$$

**Lemma 9 (see [7]).**

For any real vectors a and b and any matrix *Q* > 0 with appropriate dimensions, it follows that ±2*a* ^*T*^ *b* ≤ *a* ^*T*^ *Qa* + *b* ^*T*^ *Q* ^−1^ *b*.

## 3. Main Results

In this section, a delay-dependent leakage delay method is developed to guarantee the stochastic passive results of system (1), so we have the following Theorem 10.

**Theorem 10 .**

Given scalars *τ* > 0, 0 < *α* < 1, 0 < *β* < 1, *λ* > 0, *ρ*_*σ*_ > 0, and 0 < *μ* and proper matrix Σ_*i*_  (*i* = 1,2, 3), the SNNs described by (1) are stochastically passive in the sense of Definition 4, if there exist positive matrices *P* > 0, *Q* > 0, *Q*_*j*_ > 0  (*j* = 1,2,…, 5), and *R*_*l*_ > 0  (*l* = 1,2,…, 5), positive diagonal matrices *F*_*j*_ > 0  (*j* = 1,2), positive constants *ϵ*_1_, *ϵ*_2_, *γ* > 0, and real matrices $\bar{M}$, $\bar{N},\bar{U},\bar{S}$, and $\bar{Z}$ of appropriate dimensions such that the following LMIs hold:

(12) P+τR4≤λI,

(13) Ψi+ΩTQ−1ΩM¯N¯∗−1ατR40∗∗−11−ατR4<0,i=1,2,

(14) Ψi+ΩTQ−1ΩU¯S¯∗−1βτR40∗∗−11−βτR4<0,i=1,2,

where

(15) Ψi=Ψm×n15×15,Ψ1,1=Q1+R1+δ2R2−PA−ATP−ϵ1F1+λΣ1TΣ1+M1+M1T,Ψ1,2=−Z1A+M2T,Ψ1,3=−M1+M3T+N1,Ψ1,4=M4T−N1+U1,Ψ1,5=M5T−U1+S1,Ψ1,6=M6T−S1,Ψ1,7=ATPA+M7T,Ψ1,8=Z1W0+PW0+ϵ1F2+M8T,Ψ1,9=Z1W1+PW1+M9T,Ψ1,10=−Z1+M10T,Ψ1,11=Z1+P+M11T,Ψ1,12=M1T−M12,Ψ1,13=M13T−N1,Ψ1,14=M14T−U1,Ψ1,15=M15T−S1,Ψ2,2=Qρσ−1−ρσR1−Z2A,Ψ2,3=−ATZ3T−M2+N2,Ψ2,4=−ATZ4T−N2+U2,Ψ2,5=−ATZ5T−U2+S2,Ψ2,6=−ATZ6T−S2,Ψ2,7=−ATZ7T+ATPAρσ,Ψ2,8=−ATZ8T+Z2W0,Ψ2,9=−ATZ9T+Z2W1,Ψ2,10=−ATZ10T−ATZ3T,Ψ2,11=Z2−ATZ11T,Ψ2,12=−ATZ12T−M12,Ψ2,13=−ATZ13T−N2,Ψ2,14=−ATZ14T−U2,Ψ2,15=−ATZ15T−S2,Ψ3,3=1−ατ˙tQ2−Q1−M3−M3T+N3+N3T,Ψ3,4=−N3−M3T+N3T+U3,Ψ3,5=−M5T+N5T−U3+S3,Ψ3,6=−M6T+N6T−S3,Ψ3,7=−M7T+N6T,Ψ3,8=Z3W0−M8T+N8T,Ψ3,9=Z3W1−M9T+N9T,Ψ3,10=−Z3−M10T+N10T,Ψ3,11=Z3−M11T+N11T,Ψ3,12=−M12T+N12T−M3,Ψ3,13=−N3−M13T+N13T,Ψ3,14=−M14T+N14T−U3,Ψ3,15=−M15T+N15T−S3,Ψ4,4=1−τ˙tQ3−Q2−ϵ2F1+λΣ2TΣ2−N4−N4T+U4T+U4,Ψ4,5=−N5T+U5T−U4+S4,Ψ4,6=−N6T+U4T−S4,Ψ4,7=−N7T+U7T,Ψ4,8=Z4W0−N8T+U8T,Ψ4,9=Z4W1−N9T−ϵ2F2+U9T,Ψ4,10=−Z4−N10T+U10T,Ψ4,11=Z4−N11T+U11T,Ψ4,12=−N12T−M4+U12T,Ψ4,13=−N13T−N13+U14T,Ψ4,14=−N14T+U14T−U4,Ψ4,15=−N15T+U15T−S4,Ψ5,5=1−1−βτ˙tQ4−Q3−U5−U5T+S5+S5T,Ψ5,6=−U6T+S6T−S5,Ψ5,7=−U7T+S7T,Ψ5,8=Z5W0−U8T+S8T,Ψ5,9=Z5W1−U9T+S9T,Ψ5,10=−Z5−U10T+S10T,Ψ5,11=Z5−U11T+S11T,Ψ5,12=−U12T−M5+S12T,Ψ5,13=−U13T+S13T−N5,Ψ5,14=−U14T−U5+S14T,Ψ5,15=−U15T+S15T−S5,Ψ6,6=−Q4−S6−S6T,Ψ6,7=−S7T,Ψ6,8=Z6W0−S8T,Ψ6,9=Z6W1−S9T,Ψ6,10=−S10T−Z6,Ψ6,11=−S11TZ6,Ψ6,12=−M6−S12T,Ψ6,13=−N6−S13T,Ψ6,14=−U14−S14T,Ψ6,15=−S15T−S6,Ψ7,7=−R2,Ψ7,8=Z7W0−ATPW0,Ψ7,9=Z7W1−ATPW1,Ψ7,10=−Z7,Ψ7,11=Z7−ATP,Ψ7,12=−M7,Ψ7,13=−N7,Ψ7,14=−U7,Ψ7,15=−S7,Ψ8,8=−ϵ1I+Q5+Z8W0+W0TZ8T,Ψ8,9=W0TZ9T,Ψ8,10=W0TZ10T−Z8T,Ψ8,11=W0TZ11T−I+Z8T,Ψ8,12=W0TZ12T−M12,Ψ8,14=W0TZ14T−U8,Ψ8,13=W0TZ13T−N8,Ψ8,15=W0TZ15T−S8,Ψ9,9=−1−τ˙tQ5−Λ2I+W1TZ9T+Z9W1,Ψ9,10=W1TZ10T−Z9,Ψ9,11=Z9+W1TZ11T,Ψ9,12=−M9+W1TZ12T,Ψ9,13=−N9+W1TZ13T,Ψ9,14=−U9+W1TZ14T,Ψ9,15=−S9+W1TZ15T,Ψ10,10=Z10+Z10T+τR3,Ψ10,11=Z10−Z11T,Ψ10,12=−M10−Z12T,Ψ10,13=−Z13T−N10,Ψ10,14=−Z14T−U10,Ψ10,15=−Z15T−S10,Ψ11,11=Z11T+Z11−γI+λΣ3TΣ3,Ψ11,12=Z12T−M11,Ψ11,13=Z13T−N11,Ψ11,14=Z14T−U11,Ψ11,15=Z15T−S11,Ψ12,12=−1ατR3−M12−M12T,Ψ12,13=−M13T−N12,Ψ12,14=−M14T−U12,Ψ12,15=−M15T−S12,Ψ13,13=−11−ατR3−N13−N13T,Ψ13,14=−U13−N14T,Ψ13,15=−N15T−S13,Ψ14,14=−R31βτ−U14−U14T,Ψ14,15=−U15T−S14,Ψ15,15=−11−βτR3−S15−S15T,Ω=ρσPA00000000000000.

Ψ_1_ and Ψ_2_ are defined as replacing $\dot{\tau }(t)$ in Ψ_*i*_ by *μ* and −*μ*, respectively. Consider
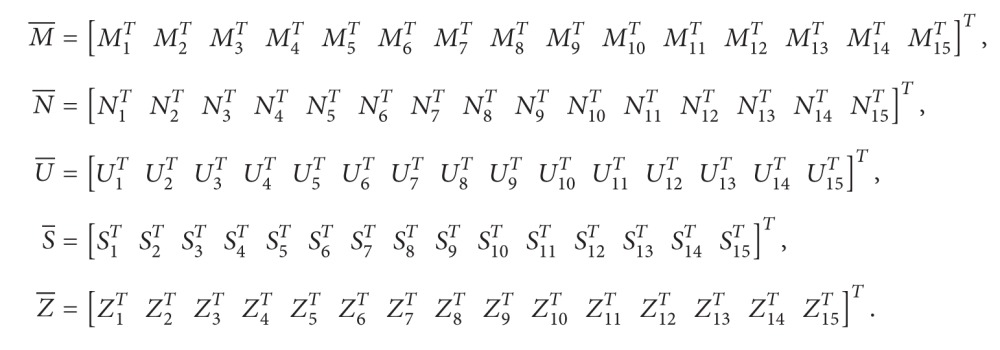
(16)

**Proof**

For the convenience of proof, we denote

(17) gt=−Axt−δt+W0fxt+W1fxt−τt+ut,σt=σxt,xt−τt,ut;

then system (1) can be rewritten as

(18) $$\begin{array}{c} dx\left(\;t\;\right)=g\left(\;t\;\right)dt+\sigma \left(\;t\;\right)d\omega \left(t\right). \end{array}$$

Choose a Lyapunov-Krasovskii functional candidate as *V*(*x*(*t*)) = ∑_*i*=1_^6^ *V*_*i*_(*x*(*t*), *t*), where

(19) V1xt,t=xt−A∫t−δttxsdsTPxt−A∫t−δttxsds,V2xt,t=∫t−δttxTsR1xTsds+δ∫−δt0∫t+θtxTsR2xTsds dθ,V3xt,t=∫t−ατttxTsQ1xsds+∫t−τtt−ατtxTsQ2xsds+∫t−φtt−τtxTsQ3xsds+∫t−τt−φtxTsQ4xsds,V4xt,t=∫t−τttfTsQ5fsds,V5xt,t=∫−τ0∫t+stgTθR3gθdθ ds,V6xt,t=∫−τ0∫t+stσTθR4σθdθ ds,

where *φ*(*t*) = *τ*(*t*) + *β*(*τ* − *τ*(*t*)) and 0 < *α* < 1 and 0 < *β* < 1. Then, the stochastic differential of *V*(*x*(*t*), *t*) along system (1) can be obtained as follows:

(20) dVxt,t=LVxt,tdt+2xtTPgtdωt,

where

(21) $$\begin{array}{c} LV\left(\;x\left(\;t\;\right),t\;\right)=\overset{6}{\underset{i=1}{\sum }}LV_{i}\left(\;x\left(\;t\;\right),t\;\right). \end{array}$$

So by Lemma 9, the following inequalities can be obtained:

(22) LV1xt,t=2xt−A∫t−δttxsdsTPg¯t+trace⁡σTtPσt≤−2xTtPAxt+2xTtPW0fxt+2xTtPW1fxt−τt+2xTtPut+2∫t−δttxsdsTATPAxt−2∫t−δttxsdsTATPW0fxt−2∫t−δttxsdsTATPW1fxt−τt+2∫t−δttxsdsTATPAxt−δtρσ−2∫t−δttxsdsTATPut+trace⁡σTtPσt+xTtPAQ−1ATPxtρσ+xTt−δtQxt−δtρσ,

where $\bar{g}(t)=-Ax(t)-\rho_{\sigma }Ax(t-\delta (t))+W_{0}f(x(t))+W_{1}f(x(t-\tau (t)))+u(t)$,

(23) LV2xt,t≤xTtR1xt−1−ρσ·xTt−δtR1xt−δt+δ2xTtR2xt−∫t−δttxTsdsR2∫t−δttxsds,LV3xt,t=xTtQ1xt+1−ατ˙t·xTt−ατtQ2−Q1xt−ατt+1−τ˙txTt−τtQ3−Q2xt−τt+1−1−βτ˙txTt−φtQ4−Q3·xt−φt−xTt−τQ4xt−τ,LV4xt,t=fTxtQ5fxt−1−τ˙t·fTxt−τtQ5fxt−τt,LV5xt,t=τgtTR3gt−∫t−ατttgTsR3gsds−∫t−τtt−ατtgTsR3gsds−∫t−φtt−τtgTsR3gsds−∫t−τt−φtgTsR3gsds,LV6xt,t=τσTtR4σt−∫t−ατttσTsR4σsds−∫t−τtt−ατtσTsR4σsds−∫t−φtt−τtσTsR4σsds−∫t−τt−φtσTsR4σsds.

By Lemma 6, it is easy to know that

(24) −∫t−ατttgTsR3gsds≤−1ατt∫t−ατttgTsdsR3∫t−ατttgsds≤−1ατ∫t−ατttgTsdsR3∫t−ατttgsds,−∫t−τtt−ατtgTsR3gsds≤−11−ατ∫t−τtt−ατtgTsdsR3∫t−τtt−ατtgsds,−∫t−φtt−τtgTsR3gsds≤−1βτ∫t−φtt−τtgTsdsR3∫t−φtt−τtgsds,−∫t−τt−φtgTsR3gsds≤−11−βτ∫t−τt−φtgTsdsR3∫t−τt−φtgsds.

For arbitrary matrices $\bar{M}$, $\bar{N}$, $\bar{U}$, $\bar{S}$, and $\bar{Z}$ with compatible dimensions, we have

(25) θ1t=2ζTtM¯xt−xTt−ατt−∫t−ατttgsds−∫t−ατttσsdws=0,

(26) θ2t=2ζTtN¯xt−ατt−xTt−τt−∫t−τtt−ατtgsds−∫t−τtt−ατtσsdws=0,

(27) θ3t=2ζTtU¯xt−τt−xTt−φt−∫t−φtt−τtgsds−∫t−φtt−τtσsdws=0,

(28) θ4t=2ζTtS¯xt−φt−xTt−τ−∫t−τt−φtgsds−∫t−τt−φtσsdws=0,

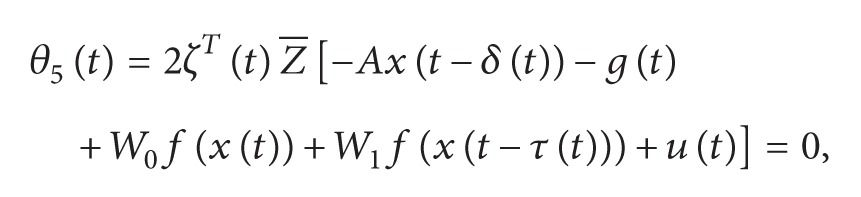
(29)where
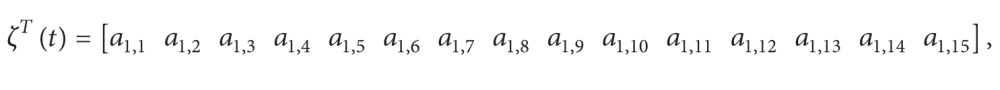
(30)where

(31) a1,1=xTt,a1,2=xTt−δt,a1,3=xTt−ατt,a1,4=xTt−τt,a1,5=xTt−φt,a1,6=xTt−τ,a1,7=∫t−δttxTsds,a1,8=fTxt,a1,9=fTxt−τt,a1,10=gTt,a1,11=uTt,a1,12=∫t−ατttgTsds,a1,13=∫t−τtt−ατtgTsds,a1,14=∫t−φtt−τtgTsds,a1,15=∫t−τt−φtgTsds.

From Assumptions 3 and (12), we can get

(32) trace⁡σTtP+τR4σt≤λxTtΣ1TΣ1xt+xTt−τtΣ2TΣ2xt−τt+uTtΣ3TΣ3ut.

In addition, from Assumption 1, the following inequalities can be deduced:

(33) fxt−Λ1xtTfxt−Λ2xt≤0,fxt−τt−Λ1xt−τtT·fxt−τt−Λ2xt−τt≤0.

It is clear that for any scalars *ϵ*_1_ > 0 and *ϵ*_2_ > 0, there exist diagonal matrices *F*_1_ ≥ 0, *F*_2_ ≥ 0, and Λ_*i*_  (*i* = 1,2) such that the following inequality hold:

(34) 0≤−ϵ1xtfxtTF1F2∗Ixtfxt=θ6t,0≤−ϵ2xt−τtfxt−τtT·F1F2∗Ixt−τtfxt−τt=θ7t,

where

(35) F1=Λ1TΛ1+Λ2TΛ12,F2=−Λ1T+Λ2T2.

In order to get the passive condition, we introduce the following inequality:

(36) LVxt,t−2yTtut−γuTtut≤LVxt,t−2fTxtut−γuTtut+∑i=17θit.

On the other hand, for formulas (25)–(28), we further have

(37) −2ζTtM¯∫t−ατttσsdws≤ζTtM¯R4−1M¯Tζt+∫t−ατttσsdwsTR4∫t−ατttσsdws,−2ζTtN¯∫t−τtt−ατtσsdws≤ζTtN¯R4−1N¯Tζt+∫t−τtt−ατtσsdwsTR4∫t−τtt−ατtσsdws,−2ζTtU¯∫t−φtt−τtσsdws≤ζTtU¯R4−1U¯Tζt+∫t−φtt−τtσsdwsTR4∫t−φtt−τtσsdws,−2ζTtS¯∫t−τt−φtσsdws≤ζTtS¯R4−1S¯Tζt+∫t−τt−φtσsdwsTR4∫t−τt−φtσsdws.

At the same time, from the character of Itô integrals, we can obtain that

(38) E∫t−ατttσTsdwsR4∫t−ατttσsdws=E∫t−ατttσTsR4σsds,

(39) E∫t−τtt−ατtσTsdwsR4∫t−τ1tt−ατtσsdws=E∫t−τtt−ατtσTsR4σsds,

(40) E∫t−φtt−τtσTsdwsR4∫t−φtt−τtσsdws=E∫t−φtt−τtσTsR4σsds,

(41) E∫t−τt−δtσTsdwsR4∫t−τt−φtσsdws=E∫t−τt−φtσTsR4σsds.

By substituting (22)-(23) into (20) and considering (36), then taking expectation on both sides of (20), and then using (38), we can get
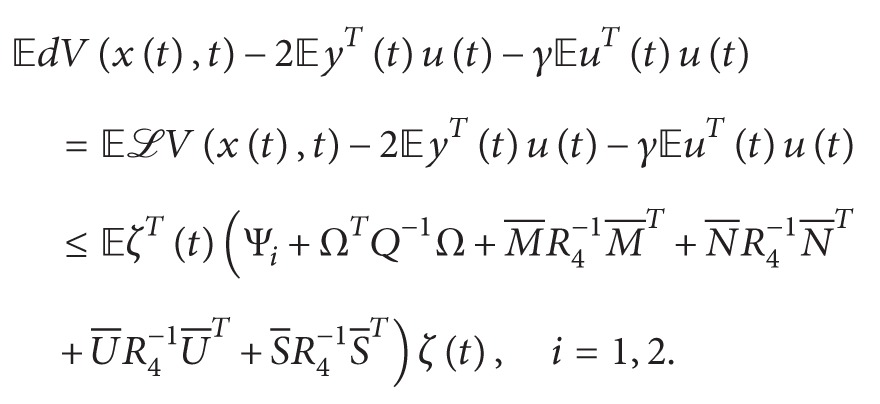
(42)By Lemma 8, there exist nonnegative functions *λ*_1_(*t*) and *λ*_2_(*t*) satisfying *λ*_1_(*t*) + *λ*_2_(*t*) = 1 such that

(43) $$\begin{array}{c} \Psi =\lambda_{1}\left(\;t\;\right)\Psi_{1}+\lambda_{2}\left(\;t\;\right)\Psi_{2}. \end{array}$$

Substituting (43) into (42), then (42) can be rewritten as

(44) EdVxt,t−2EyTtut−γEuTtut≤λ1tθ1tζTt·Ψ1+ΩTQ−1Ω+MR4−1MT+NR4−1NTζt+λ1tθ2tζTt·Ψ2+ΩTQ−1Ω+MR4−1MT+NR4−1NTζt·λ2tθ1tζTt·Ψ1+ΩTQ−1Ω+UR4−1UT+SR4−1STζt+λ2tθ2tζTt·Ψ2+ΩTQ−1Ω+UR4−1UT+SR4−1STζt,

where *θ*_1_(*t*) = *τ*(*t*)/*τ* ≥ 0,  *θ*_2_(*t*) = (*τ* − *τ*(*t*))/*τ* ≥ 0.

So we can get that the following matrix inequalities hold:

(45) Ψi+ΩTQ−1Ω+M¯R4−1M¯T+N¯R4−1N¯T<0,i=1,2,

(46) Ψi+ΩTQ−1Ω+U¯R4−1U¯T+S¯R4−1S¯T<0,i=1,2.

By virtue of Lemma 7, (45) and (46) are equivalent to (13) and (14), respectively, so we can get that

(47) $$\begin{array}{c} EdV\left(\;x\left(\;t\;\right),t\;\right)-2Ey^{T}\left(\;t\;\right)u\left(\;t\;\right)-\gamma Eu^{T}\left(\;t\;\right)u\left(\;t\;\right)<0; \end{array}$$

then integrating on both sides of (47) from 0 to *t*, we can obtain

(48) 2E∫0tyTsusdsEVxt,t−EVx0,0−γE∫0tususds≥−γE∫0tuTsusds.

It indicates that system (1) is stochastically passive in the sense of Definition 4. This completes the proof.

**Remark 11 .**

In [42], the delay interval is divided into three subintervals, which are [−*τ*_2_, −*τ*(*t*)], [−*τ*(*t*), −*τ*_1_], and [−*τ*_1_, 0]. In this paper, the new Lyapunov function proposed in Theorem 10 is based on the decomposition of delay interval [−*τ*, 0] into four subintervals, which are [−*ατ*(*t*), 0], [−*τ*(*t*), −*ατ*(*t*)], [−*φ*(*t*), −*τ*(*t*)], and [−*τ*, −*τ*(*t*)]. By using the lower bound and upper bound of delay derivative $\dot{\tau }(t)$, the idea of delay fraction can be successfully applied to cases of both constant and time-varying delay, so less conservatism results can be expected.

When the leakage delay is constant, namely, *δ*(*t*) = *δ* and *ρ*_*σ*_ = 0, neural network system (1) will become the following model:

(49) dxt=−Axt−δ+W0fxt+W1fxt−τt+utdt+σxt,xt−τt,utdωt,yt=fxt.

This system has been studied in [42]; then for system (49) we have the following Corollary 12.

**Corollary 12 .**

Given scalars *τ* > 0, 0 < *α* < 1, 0 < *β* < 1, *λ* > 0, and 0 < *μ* and proper matrices Σ_*i*_  (*i* = 1,2, 3), the SNNs described by (49) are stochastically passive in the sense of Definition 4, if there exist positive matrices *P* > 0, *Q*_*j*_ > 0  (*j* = 1,…, 5), and *R*_*l*_ > 0  (*l* = 1,…, 5), positive diagonal matrices *F*_*j*_ > 0(*j* = 1,2), positive constants *γ* > 0, *ϵ*_1_, *ϵ*_2_, and real matrices $\bar{M},\bar{N},\bar{U},\bar{S}$, and $\bar{Z}$ of appropriate dimensions such that the following LMIs hold:

(50) P+τR4≤λI,

(51) Ψ¯iτ˙tMN∗−1ατR40∗∗−11−ατR4<0,i=1,2,

(52) Ψ¯iτ˙tUS∗−1βτR40∗∗−11−βτR4<0,i=1,2,

where

(53) Ψ¯2,2=−R1−Z2A,Ψ¯2,7=0.

${\bar{\Psi }}_{1}(\dot{\tau }(t))$ and ${\bar{\Psi }}_{2}(\dot{\tau }(t))$ are defined as replacing $\dot{\tau }(t)$ in ${\bar{\Psi }}_{i}(\dot{\tau }(t))$ by *μ* and −*μ*, respectively; the other terms have the same expression as that in Theorem 10.

It is well known that the Markovian jump systems (MJSs) are a special class of hybrid systems, which have the advantage in modeling the dynamic systems subject to abrupt variation in their structures, such as component failures and sudden environmental disturbance. Many researches about the stability analysis, impulsive response, and state estimation on the neural networks with Markovian jumping parameters have been obtained; see [44–47] and references therein. Recently [48] has studied the passivity of stochastic neural networks with Markovian jumping parameters; the same method can be used to a system with Markovian jumping parameters and it still leaves much room to reduce the conservatism, which motivates our aim.

Let *r*_*t*_, *t* ≥ 0, be a right-continuous Markov chain defined on a complete probability space (*Ω*, *ℱ*, *P*) and taking discrete values in a finite state space *S* = {1,2,…, *N*} with generator Π = (*π*_*ij*_)_*N*×*N*_ given by

(54) Prt+Δ=j ∣ rt=i=πijΔ+oΔ,i≠j,1+πijΔ+oΔ,i=j,

where Δ > 0 and *π*_*ij*_ ≥ 0 is the transition rate from *i* to *j* while *π*_*ii*_ = −∑_*j*≠*i*_ *π*_*ij*_.

For the purpose of simplicity, in the sequel, for each *r*_*t*_ = *i* ∈ *S*, *A*(*r*_*t*_), *W*_0_(*r*_*t*_), and *W*_1_(*r*_*t*_) are denoted by *A*_*i*_, *W*_0*i*_, *W*_1*i*_, and so on. Throughout the paper, we assume that *ω*(*t*) and *r*(*t*) are independent. Then when the leakage delay does not exist, system (1) will become the one as follows:

(55) dxt=−Aixt+W0ifxt+W1ifxt−τt+utdt+σxt,xt−τt,i,utdωtyt=fxt.

This system has been studied in [43] and good results have been obtained. In order to testify the effectiveness of our methods, we give the following Theorem 13.

**Theorem 13 .**

Given scalars *τ* > 0, 0 < *α* < 1, 0 < *β* < 1, *λ*_*i*_ > 0, and 0 < *μ* and proper matrices Σ_*n*_  (*n* = 1,2, 3), the SNNs described by (1) are stochastically passive in the sense of Definition 4, if there exist positive matrices *P*_*i*_ > 0, *Q* > 0, *Q*_*j*_ > 0  (*j* = 1,…, 5), and *R*_*l*_ > 0  (*l* = 1,2,…, 5), positive diagonal matrices *F*_*j*_ > 0(*j* = 1,2), positive constants *ϵ*_1*i*_, *ϵ*_2*i*_, *γ* > 0, and real matrices ${\bar{M}}_{i},{\bar{N}}_{i},{\bar{U}}_{i},{\bar{S}}_{i}$, and ${\bar{Z}}_{i}$ of appropriate dimensions such that the following LMIs hold:

(56) Pi+τR4≤λiI,

(57) Ψκτ˙tM¯iN¯i∗−1ατR40∗∗−11−ατR4<0,κ=1,2

(58) Ψκτ˙tU¯iS¯i∗−1βτR40∗∗−11−βτR4<0,κ=1,2,

where

(59) Ψiτ˙t=Ψm×n13×13,Ψ1,1=Q1+R1+∑j=1NπijPj−PiAi−AiTPi−ϵ1iF1+λiΣ1iTΣ1i+M1i+M1iT,Ψ1,2=−M1i+M2iT+N1i,Ψ1,3=M3iT−N1i+U1i,Ψ1,4=M4iT−U1i+S1i,Ψ1,5=M5iT−S1i,Ψ1,6=Z1iW0i+PW0i+ϵ1iF2+M6iT,Ψ1,7=Z1iW1i+PiW1i+M7iT,Ψ1,8=−Z1i+M8iT,Ψ1,9=Z1i+P+M9iT,Ψ1,10=M1iT−M10i,Ψ1,11=M11iT−N1i,Ψ1,12=M12iT−U1i,Ψ1,13=M13iT−S1i,Ψ2,2=1−ατ˙tQ2−Q1−M2i−M2iT+N2i+N2iT,Ψ2,3=−N2i−M2iT+N2iT+U2i,Ψ2,4=−M4iT+N4iT−U2i+S2i,Ψ2,5=−M5iT+N5iT−S2i,Ψ2,6=Z2iW0i−M6iT+N6iT,Ψ2,7=Z2iW1i−M7iT+N7iT,Ψ2,8=−Z2i−M8T+N8T,Ψ2,9=Z2i−M9iT+N9iT,Ψ2,10=−M10iT+N10iT−M2i,Ψ2,11=−N2i−M11iT+N11iT,Ψ2,12=−M12iT+N12iT−U2i,Ψ2,13=−M13iT+N13iT−S2i,Ψ3,3=1−τ˙tQ3−Q2−ϵ2iF1+λΣ2iTΣ2i−N3i−N3iT+U3iT+U3i,Ψ3,4=−N4iT+U4iT−U3i+S3i,Ψ3,5=−N5iT+U3iT−S3i,Ψ3,6=Z3iW0i−N6iT+U6iT,Ψ3,7=Z3iW1i−N7T−ϵ2iF2+U7iT,Ψ3,8=−Z3i−N8iT+U8iT,Ψ3,9=Z3−N9T+U9T,Ψ3,10=−N10T−M3+U10T,Ψ3,11=−N11iT−N11i+U11iT,Ψ3,12=−N12iT+U12iT−U3i,Ψ3,13=−N13iT+U13iT−S3i,Ψ4,4=1−1−βτ˙tQ4−Q3−U4i−U4iT+S4i+S4iT,Ψ4,5=−U5iT+S5iT−S4i,Ψ4,6=Z4iW0i−U6iT+S6iT,Ψ4,7=Z4iW1i−U7iT+S7iT,Ψ4,8=−Z4i−U8iT+S8iT,Ψ4,9=Z4i−U9iT+S9iT,Ψ4,10=−U10iT−M4i+S10iT,Ψ4,11=−U11iT+S11iT−N4i,Ψ4,12=−U12iT−U4i+S12iT,Ψ4,13=−U13iT+S13iT−S4i,Ψ5,5=−Q4−S5i−S5iT,Ψ5,6=Z5iW0i−S6iT,Ψ5,7=Z5iW1i−S7T,Ψ5,8=−S8iT−Z5i,Ψ5,9=−S9iTZ5i,Ψ5,10=−M5i−S10iT,Ψ5,11=−N5i−S11iT,Ψ5,12=−U12i−S12iT,Ψ5,13=−S13iT−S5i,Ψ6,6=−ϵ1iI+Q5+Z6iW0i+W0iTZ6iT,Ψ6,7=W0iTZ7iT,Ψ6,8=W0iTZ8iT−Z6T,Ψ6,9=W0iTZ9iT−I+Z6iT,Ψ6,10=W0iTZ10iT−M10i,Ψ6,12=W0iTZ12iT−U6i,Ψ6,11=W0iTZ11iT−N6i,Ψ6,13=W0TZ13T−S6,Ψ7,7=−1−τ˙tQ5−ϵ2iI+W1iTZ7iT+Z7iW1i,Ψ7,8=W1iTZ8iT−Z7i,Ψ7,9=Z7i+W1TZ9iT,Ψ7,10=−M7i+W1iTZ10iT,Ψ7,11=−N7i+W1iTZ11iT,Ψ7,12=−U7i+W1iTZ12iT,Ψ7,13=−S7i+W1iTZ13iT,Ψ8,8=Z8i+Z8iT+τR3,Ψ8,9=Z8i−Z9iT,Ψ8,10=−M8i−Z10iT,Ψ8,11=−Z11iT−N8i,Ψ8,12=−Z12iT−U8i,Ψ8,13=−Z13iT−S8i,Ψ9,9=Z9iT+Z9i−γI+λiΣ3iTΣ3i,Ψ9,10=Z10iT−M9i,Ψ9,11=Z11iT−N9i,Ψ9,12=Z12iT−U9i,Ψ9,13=Z3iT−S9i,Ψ10,10=−1ατR3−M10i−M10iT,Ψ10,11=−M11iT−N10i,Ψ10,12=−M12iT−U10i,Ψ10,13=−M13iT−S10i,Ψ11,11=−11−ατR3−N11i−N11iT,Ψ11,12=−U11i−N12iT,Ψ11,13=−N13iT−S11i,Ψ12,12=−R31βτ−U12i−U12iT,Ψ12,13=−U13iT−S12i,Ψ13,13=−11−βτR3−S13i−S13iT.

$\Psi_{1}(\dot{\tau }(t))$ and $\Psi_{2}(\dot{\tau }(t))$ are defined as replacing $\dot{\tau }(t)$ in $\Psi_{i}(\dot{\tau }(t))$ by *μ* and −*μ*, respectively. One has
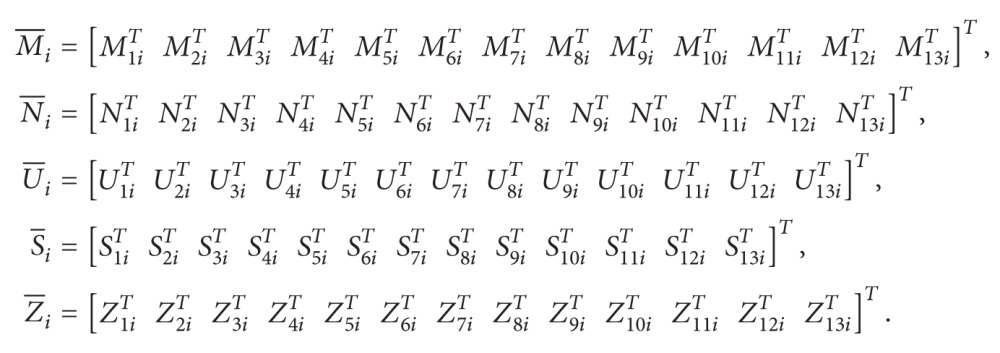
(60)

**Proof**

Choose a Lyapunov-Krasovskii functional candidate as *V*(*x*(*t*), *t*, *i*) = ∑_*N*=1_^5^ *V*_*N*_(*x*(*t*), *t*, *i*), where

(61) V1xt,t,i=xtTPixt,V2xt,t,i=∫t−ατttxTsQ1xsds+∫t−τtt−ατtxTsQ2xsds+∫t−φtt−τtxTsQ3xsds+∫t−τt−φtxTsQ4xsds,V3xt,t,i=∫t−τttfTsQ5fsds,V4xt,t,i=∫−τ0∫t+stgTθR3gθdθ ds,V5xt,t,i=∫−τ0∫t+stσTθR4σθdθ ds,

where *φ*(*t*) = *τ*(*t*) + *β*(*τ* − *τ*(*t*)) and 0 < *α* < 1 and 0 < *β* < 1.

By the same method as that in Theorem 10, we can get that the following inequalities hold:
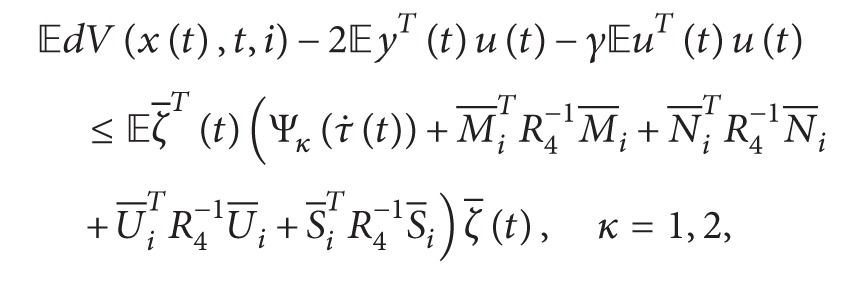
(62)where
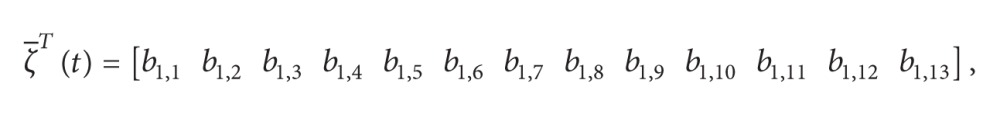
(63)where

(64) b1,1=xTt,b1,2=xTt−ατt,b1,3=xTt−τt,b1,4=xTt−φt,b1,5=xTt−τ,b1,6=fTxt,b1,7=fTxt−τt,b1,8=gTt,b1,9=uTt,b1,10=∫t−ατttgTsds,b1,11=∫t−τtt−ατtgTsds,b1,12=∫t−φtt−τtgTsds,b1,13=∫t−τt−φtgTsds.

So by virtue of Lemma 7 and the same proof method of Theorem 10, we can get that system (55) is stochastic passive.

## 4. Numerical Example and Simulation

In this section, three numerical examples are presented to demonstrate the effectiveness of the developed method on the obtained passive results.

**Example 1 .**

Consider neutral stochastic neural networks (1) with the following parameters (Example  5 in [42]):

(65) A=1.5001.3,W0=0.50.20.40.3,W1=0.4−0.10.10.2,Σ1=0.1000.1,Σ2=0.2000.2,Σ3=0.3000.4,α=0.5,β=0.6,τ=0.8,μ=0.6.

Take the activation function as *f*_1_(*x*(*t*)) = *f*_2_(*x*(*t*)) = tanh⁡(*x*), so it can be verified from Assumption 1 that *F*_1_ = diag⁡{0,0} and *F*_2_ = diag⁡{−0.5, −0.5}, and by using of the Matlab LMI Control Toolbox, we find out a solution to LMIs (12), (13), and (14) as follows:

(66) P=0.1536−0.0046−0.00460.1497,R1=0.0812−0.0415−0.04150.0864,R2=1.4662−0.4043−0.40431.2144,R3=0.0088−0.0058−0.00580.0122,R4=0.05200.00510.00510.0565,Q1=0.0881−0.0174−0.01740.0902,Q2=0.0704−0.0139−0.01390.0717,Q3=0.0529−0.0102−0.01020.0537,Q4=0.0318−0.0060−0.00600.0322,Q=0.0318−0.0060−0.00600.0322,γ=297.6609.

In order to testify the effectiveness of our proposed method, many experiments have been done and the upper bounds of delays *δ* and *τ* are listed from Tables 1 to 3, where “–” means that LMIs (12)–(14) has no feasible solution.  Table 1 shows the maximum allowable upper bound *δ* for different values of *ρ*_*σ*_, which means that the bound of the derivative of the leakage-time-varying is very effective and plays an important role in obtaining the feasible results.

From Table 2, we can see that when *μ* > 1, the feasible solution can be obtained. From Table 3 we can see that when fixing the value of *μ* and *ρ*_*σ*_, the allowable upper value of *τ* is effected by *δ*, especially when *δ* = 0.45; the feasible solution cannot be obtained. Especially, when leakage *δ*(*t*) = *δ*, namely, leakage delay, is constant, the studied system will become system (40), which has been researched in [42]; then we have the following Example 2.

**Example 2 .**

Consider that stochastic neural networks (49) have the same parameters as that in [42], so from Corollary 12, we can have the following research results listed in Tables 4 and 5, which show the effect on *τ* for different *μ* and mutual effect between *τ* and *δ*.

From Table 4 we also can see that when the same parameters in (2) and (3) of [42] are taken into account and when *λ* is set by 1 and *δ* = 0.1 and *R*_1_ = *R*_2_ = 0.1*∗I*, by solving (2) and (3) of [42], we can get that maximum value *τ* is 0.0005, so our method has obtained the less conservatism than that of [42].

**Figure 1:**
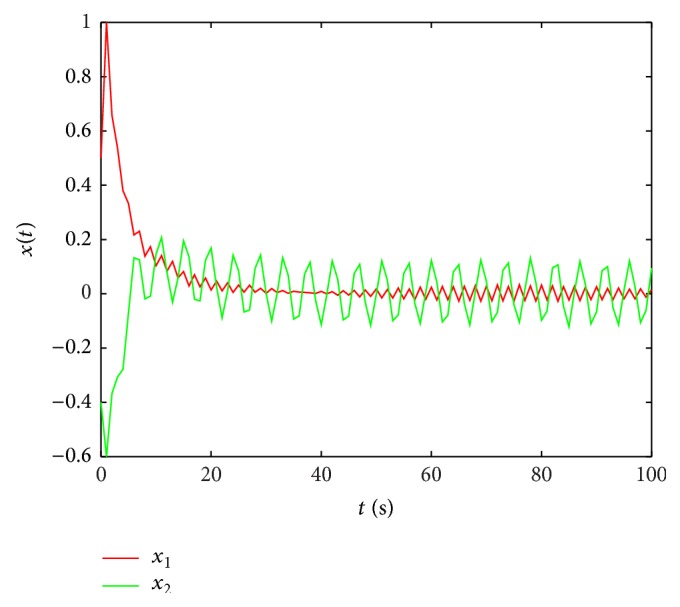
State curves of system (1) with input *u*(*t*).

In this example, when $u\left(t\right)={\left[\begin{array}{cc} -0.3\cos\left(3.1t\right) & 0.7\sin\left(1.4t\right) \end{array}\right]}^{\prime }$, Figure 1 shows the state curve with *u*(*t*). From Figure 1 we can see that when stochastic disturbance and input exist, the systems with leakage delay are unstable.

**Remark 14 .**

In [42], the sufficient conditions of passivity about stochastic neural networks are given by LMIs, but the solution is not given out, and the simulation about both stochastic and leakage delay is not discussed, either. In our discussion, the impact of leakage delay on stability of systems is considered.

**Figure 2:**
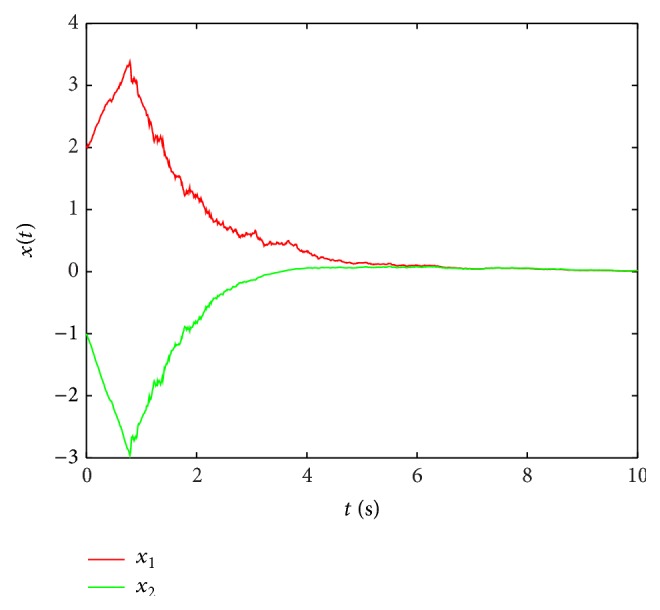
State curves of system (1) without input and *δ* is 0.6.

**Figure 3:**
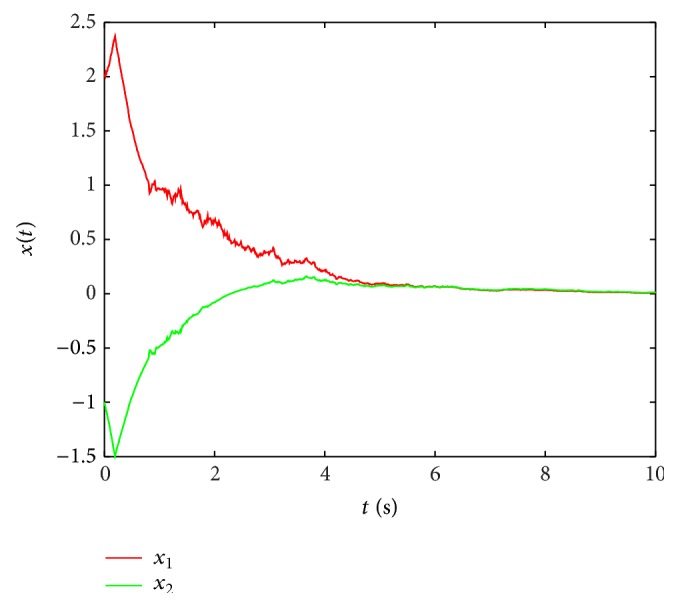
State curves of system (49) with leakage delay 0.2.

At the same time, when leakage delay is set by different values, by taking the initial state [2, −1] and using the Matlab software, a state curve is obtained as in Figure 2; Figure 2 shows the state curves of system (49) without input and *δ* is 0.6 and *τ* = 0.8; Figure 3 shows the state curves of system (49) without input and *δ* is 0.2 and *τ* = 0.8.

**Figure 4:**
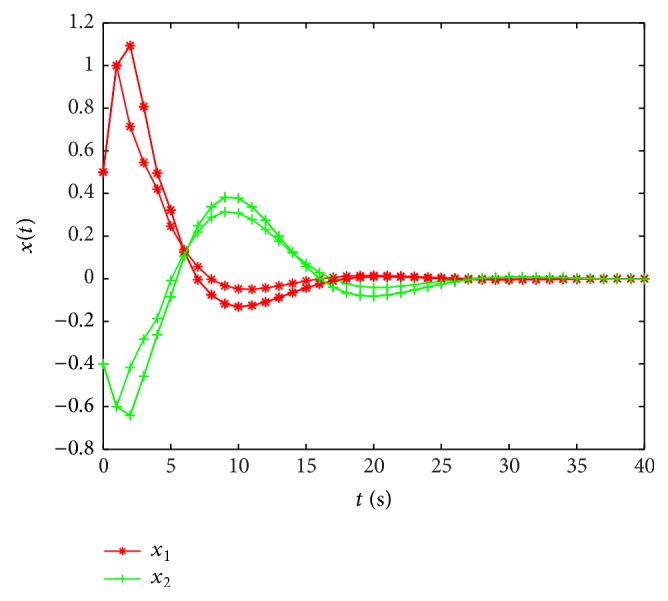
State curves of system (1) without input.

When stochastic disturbance does not exit in system (49), the state simulation curves of (49) are shown in Figure 4.

Figure 4 shows the state curves of system (49) without stochastic disturbance and different leakage delay; from Figure 4, we can see that when the leakage delay exits in the neural networks system, state curve of system oscillates sharply from the start point and then becomes asymptotically stable; at the same time, we can find that the bigger the leakage delay, the more serious the oscillation.

**Remark 15 .**

In Corollary 2 of [42], the maximum value of time delay is 0.2, when leakage delay is set by 0.1. In our method, combing the simulation curve with the value of *δ*, when *δ* is 0.1, the maximum value of time delay which is guaranteeing the fact that system (1) is stable can reach 1.2.

**Remark 16 .**

In [42], an example has been given to show the effectiveness of passivity criteria, but how the leakage delay affects the stability is not discussed. In our example, simulations have been given and proved that leakage delay can cause effect on the stability of neural networks.

**Example 3 .**

Consider a two-neuron stochastic neural network with Markovian jump parameters and mixed time delays (56) with the following parameters [43]:

 Mode 1
  (67) A1=4003,W01=0−0.50.50,W1=0.4−0.50.50,Σ11=0.2000.5,Σ12=0.5000.2,Σ13=0.3000.2.
 Mode 2
  (68) A2=3004.5,W02=01−11,W12=−1−11−2,Σ21=0.4000.2,Σ22=0.2000.3,Σ23=0.2000.4.

Let the Markov process governing the mode switching have generator

(69) $$\begin{array}{c} \prod =\left[\begin{array}{cc} -1 & 1\\ 0.5 & -0.5 \end{array}\right]. \end{array}$$

We take *f*_*j*_(*x*_*j*_) = tanh⁡(*x*_*j*_), *α* = 0.5, *μ* = 0.5, *β* = 0.6, and *τ*(*t*) = 0.7 + 0.1cos⁡(*t*); by solving the LMI in Theorem 13, the following feasible solutions can be obtained:

(70) P1=112.9775−8.7790−8.7790148.8517,P2=143.0854−14.0663−14.0663105.4530,Q1=357.2075−34.0926−34.0926340.8987,Q2=246.3834−36.6553−36.6553210.3547,Q3=117.8358−22.8272−22.8272152.6593,Q4=105.2710−9.7393−9.7393117.4932,Q5=144.194617.597617.5976181.2422,R1=73.0134−23.6982−23.698286.0642,R3=48.8051−13.1574−13.157425.3595,R4=221.037810.834710.8347212.3304,γ=414.5897,λ1=416.0385,λ2=437.0104,ϵ1=434.0883,ϵ2=184.0411.

At the same time, in order to testify the less conservatism of our method, the allowable upper bounds of *τ* with different values of *μ* have been compared with that in [43]; the results are shown in Table 6.

From Table 6, we can see that even *μ* > 1; our methods can improve existing research results.

**Figure 5:**
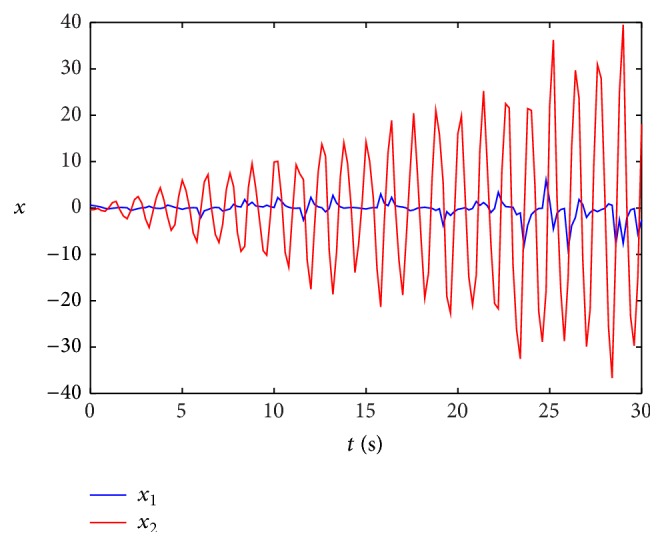
State curves of system (55) with input.

**Figure 6:**
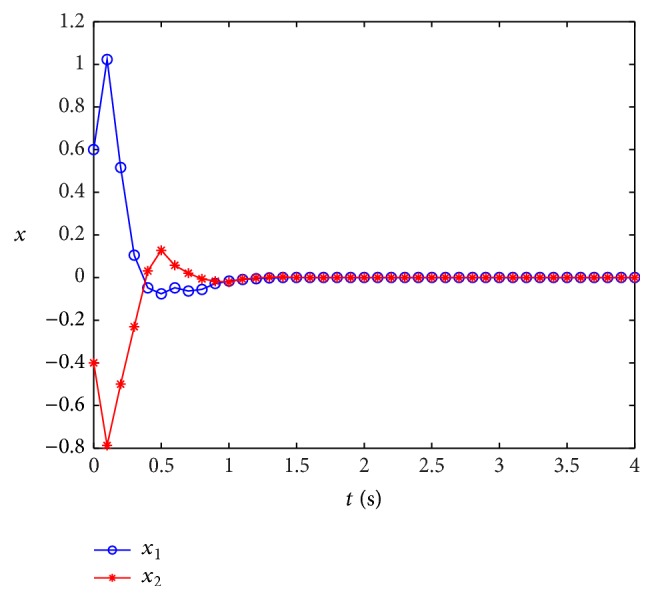
State curves of system (55) without input.

**Figure 7:**
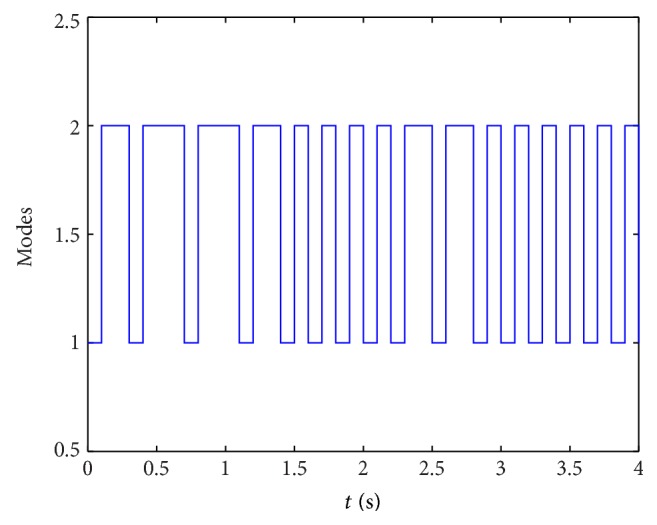
The Markov chain of system (55).

On the other hand, we select *x*(0) = [0.6, −0.4]^*T*^ and *u*(*t*) = [sin⁡(*t*), *t∗*cos⁡(*t*)]^*T*^, and the following simulation results can be obtained.  Figure 5 shows the state curve of system (56) with *u*(*t*), Figure 6 shows the state curve of system (56) without *u*(*t*), and Figure 7 shows the state switching modes of system (56), so the simulation results further prove that the two-neuron stochastic neural networks with Markovian switching parameters is passive in the sense of Definition 4.

## 5. Conclusions

In this paper, we have investigated the passivity problem for a class of stochastic neural networks systems (SNNs) with varying delay and leakage delay. By constructing a novel Lyapunov functional and utilizing the delay fractionizing technique, new passivity conditions have been established to achieve the passivity performance. Moreover, in derivation of the passivity criteria, it is assumed that the description of the activation functions is more general than the commonly used Lipschitz conditions; the time-varying delay is divided into several subintervals; two adjustable parameters are introduced so that more information about time delay has been utilised. Finally, examples and simulations are provided to illustrate the impact of leakage delay on stability of neural networks and the less conservatism of the developed approach.

## Figures and Tables

**Table 1:** Maximum allowable bounds of *δ* for different *ρ*_*σ*_ values and *α* = 0.6, *β* = 0.6, *τ* = 1.0, and *μ* = 0.6.

*ρ*_*σ*_	0.1	0.4	0.45
Theorem 10	0.5176	0.3251	—

**Table 2:** Maximum allowable bounds of *τ* for different *μ* values of *ρ*_*σ*_ = 0.2 and *δ* = 0.2.

*μ*	0	0.5	0.8	1.1
*τ*	0.8267	0.7342	0.4682	0.3848

**Table 3:** Allowable upper bounds of *τ* for different values of *δ*; *ρ*_*σ*_ = 0.1 and *μ* = 0.5.

*δ*	0.1	0.05	0.1	0.4	0.45
*τ*	0.8367	0.7537	0.6415	0.2593	—

**Table 4:** Allowable upper bounds of *τ* for different values of *δ*; *μ* = 0.5.

*δ*	0.01	0.05	0.1	0.4	0.45
Corollary 12	0.9293	0.7882	0.7023	0.3685	—

[42]	0.0005	—	—	—	—

**Table 5:** Allowable upper bounds of *τ* with different values of *μ*; *α* = 0.4, *β* = 0.6, and *δ* = 0.2.

*μ*	0	0.2	0.5	0.8	0.9	1.1
*τ*	0.3025	0.3004	0.2946	0.2886	0.2886	0.2886

**Table 6:** Allowable upper bounds of *τ* with different values of *μ*; *α* = 0.5 and *β* = 0.6.

*μ*	0	0.4	0.8	1.1
[43]	0.3025	0.2946	0.1886	0.1826

Theorem 13	0.8226	0.6357	0.4786	0.2839
